# What Can Oral Prophylaxis and Orthodontia Do for Each Other?

**Published:** 1909-01-15

**Authors:** N. S. Hoff


### WHAT CAN ORAL PROPHYLAXIS AND ORTHODONTIA DO FOR EACH OTHER.?

BY N. S. HOFF, D.D.S.

I shall try to present three propositions that you may determine whether oral prophylaxis has any relation to them and the principles or practice of orthodontia. In this presentation I shall claim the privilege of utilizing all operative and prosthetic procedures which may be employed in securing complete oral prophylaxis.

First. Can oral prophylaxis as at present practiced be made an important factor in the normal development of the teeth and their surrounding structures, rendering orthodontic interference unnecessary or less difficult.

I shall not take much of your time to remind you that one of the ordinary principles of your art should be to begin your work at the earliest period practicable, and that like all other branches of the healing art, prevention of deformities is as much a department of your work as correction. If this premise is acceptable, you should enlarge your sphere of influence to include the supervision of the first, as well as the succeeding dentitions. Modesty may cause some of you to hesitate to claim this, but I predict that in the near future your best work will be done in conferring with mothers of infants as to the best measures to be adopted to secure a normal development of the teeth and jaws of the first dentition.

By all the principles of logic and reason this work ought to fall to the lot of the orthodontist, since it is his business to not only correct the mistakes of nature, but to present to the practitioner of surgical and prosthetic dentistry the teeth in a perfect state of development as possible. The business of the general practitioner of dentistry is with the prevention and repair of the ravages of disease of the teeth and their enviroment.

It is only recently that we have begun to notice that many deciduous dentures are abnormal as to development of the teeth and their positions in the arches. Very few orthodontists, however, have had the courage to suggest or have offered to apply treatment for their correction. It is generally considered hardly worth while to take the trouble to interfere with these conditions, considering their brief existence. But when we come to consider the influence these deciduous dentures have in casting the form as well as structure of the more durable second denture, I believe that we will conclude that it is well worth our while to make sure that the way for the normal development of the permanent denture is made as clear and easy as possible.

To some extent we have tried to educate people to take care of the first dentition by prescribing a suitable mouth toilet, but this is usually so carelessly done by the dentist that it makes no serious impression on the mother, and nothing adequate is done until some of the deciduous teeth begin to decay and the child complains of tooth ache when he eats candy. He is then taken to the dentist, who says he will fill that tooth with a little cement or amalgam which will stop the ache until the tooth is replaced by the permanent one. Is'nt this the practice generally? There may be a few dentists who are somewhat more thoughtful and take pains to repair damages in such a way as to not only conserve the tooth, but to completely restore its function.

I am not able to supply statistics which would prove beyond doubt that many cases of malocclusion in the permanent dentures are largely the result of lack of proper care of the deciduous dentures, but I cannot help believing that if more pains were taken to secure perfect deciduous dentures and to keeping them in perfect condition until the proper time for their removal had arrived, that we should have less malocclusion of the permanent dentures.

But what has oral prophylaxis to offer that will encourage the orthodontist to begin his work at a period where he should be able to produce the most perfect results?

When we read Dr. Smith's statements a few years ago to the effect that by periodically polishing the teeth and by regular and systematic care by the patient he was able to remove pigments that seemed permanently established in the enamel, and that there was a new life force in the pulp and peridental structures, we thought he was a dreaer,m and we had little faith to believe. After seeing actual results in a large number of cases, we became convinced that there was truth in his statements and we were encouraged to experiment for ourselves. Three years of practice and observation have convinced us that much can be accomplished by the so called prophylaxis measures in restoring the vital functions of the oral tissues, by systematic massage and cleanliness in the mouths of patients far past the constructive age in life. More recently it has come to us that if old and diseased tissues can be revived by a carefully applied system of cleanliness and stimulation why should not a similar treatment be valuable in encouraging a better development of the growing structures. We have as yet not gone back as far as the cradle, but we are working in that direction. In the few cases that we have had under observation we are confident that an early application of the system of oral prophylaxis has had the most beneficial influence, not only in retarding and preventing decay of the teeth, but we believe a good influence towards better development of the permanent teeth and jaws has resulted. Of course, the time and opportunity at our disposal has been insufficient to enable us to make positive statements, but I really believe that by careful application of the principles of oral prophylaxis, beginning with the eruption of the first deciduous tooth, it would be entirely practicable to carry any child through to complete permanent dentition without other surgical or orthodontic interference. Such a proceeding might interfere with our present business methods and practice, but I am sure our efforts would be more cordially appreciated and our art greatly enobled. A research to prove this hypothesis would be difficult, because of the time necessarily involved, as well as other obvious conditions. Its results, however, would be of immense value, and it is a point that I trust you may keep under observation.

There is no question, however, but that while cases are under treatment some more adequate method of cleanliness and stimulation should be found. The expedients now used for cleaning the teeth and appliances of incidental and extraneous deposits are obviously necessary, but insufficient. A systematic application of oral prophylaxis, that is the polishing of the teeth and stimulation of the gums and peridental membrane at frequent intervals, should encourage the formation of new repair tissue and render retention by mechanical fixtures less obligatory. The appliances at present in use render this somewhat difficult, but by keeping in mind the necessity for this treatment, it is not difficult to devise methods of applying bands, arches and ligatures in such a way as to accomplish needed movements and at the same time leave opportunity for the proper application of the prophylaxis treatment, and the regular use by the patient of the brush and powder, or disinfecting mouth washes. But this leads us to the second division of our subject.

Secondly—Can prophylaxis methods offer anything helpful during treatment for correction of maloccusion?

Every one who has practiced orthodontia using the various forms of fixed appliances, has been compelled to devise new ways of cleaning the teeth and appliances, and while many effective methods are now in use, a careful inspection of most mouths undergoing treatment will reveal diseased conditions of the gums, if not incipient caries, in spite of all efforts at cleanliness by the patient and operator. We are not sure that modern orthodontic appliances can be indicted seriously because of the unsanitary conditions they create. With these appliances the regular cleansing of the teeth by the usual tooth brush and powder in many instances is impracticable, and resort is had to antiseptic mouth washes, because they can be applied without danger or disturbing the delicately adjusted ligatures and arches. While these are helpful and convenient for use, they are in most cases insufficient. We were greatly interested in the presentation of the method of sterilization made by Dr. Ferris at the meeting last year, and trust that in the discussion those who have made considerable use of it will report results. The method is undoubtedly effective, but can never be sufficient, because it cannot be applied by the patient, or conveniently at sufficiently frequent intervals to secure the best results. Another objection to depending upon such a measure is that it does not thoroughly remove the material which is disinfected, nor leave the appliances in the best condition to prevent subsequent deposits. Too frequent application of this remedy, like other liquid disinfectants used in the mouth, leads to serious irritation of the mucous membrane and disasterous results. It has been found that the less chemical remedies used in the prophylaxis treatment of the gums, the better the healing process takes place, and we shall find that although good disinfection and a degree of cleansing is produced by forcibly spraying these alkaline chemicals into the mouth, that, unless great care is exercised, injurious, rather than beneficial results will follow. The great difficulty which confronts us is due to the celerity with which reinfection takes place, because of the exposure inevitable in the mouth, which fortunately is to some extent counteracted by the restraining influence of the saliva when it is in a normal condition,--and the practical impossibility of sufficiently frequent application of antiseptic solution to control the tendency to recurrent unhygienic conditions. We are not prepared to offer a satisfactory solution of this problem, but believe that much can be done to prevent unfavorable results by means that we can devise and control and which may be put into the hands of the patients with safety.

In the first place we would suggest that before any case of malocclusion is put under treatment the mouth should be given a thorough prophylaxis treatment, including the filling of all decayed teeth with a durable material and in a thorough manner. The appliance should be devised with an idea of making it practicable for the patient to accomplish thorough cleanliness by the frequent use of ordinary mouth toilet preparations. All bands should be accurately adjusted, the arches fitted so they will withstand considerable manipulation by a tooth brush without losing their adjustment and effectiveness. The ligatures especially should be adjusted firmly and the free ends secured so they will not become loosened or serve as places for the collection of deposits of food, etc., and above all every tooth should be approachable for thorough polishing on all of its exposed surfaces. The bands, arches and ligatures should be rendered cleanable. This can be accomplished in almost every case where metal appliances are employed. The rubber bands, silk or linen ligatures are unsanitary and need frequent renewals if used, as they are incapable of sterilization; threaded arches are objectionable as they afford lodgement for deposits; in fact, all the appliances and adjustments should be made as nearly self cleansing as practicable. Once each week, or oftener if necessary, the mouth should have a prophylaxis treatment, and every unclean spot pointed out to the patient for especial attention. The patient's duty should be to keep the mouth clean during the intervals of absence from the office by such means as may be effective. We find the best means is by the constant use of a good tooth powder and a soft brush. A powder should be provided that is not strongly akaline or in other ways much medicated, and is abrasive and yet soluble. The following formula will serve as a basis and can be modified to meet varying conditions.

| | | | |
|---|---|---|---|
| R | Precipitated chalk - - - | Parts | 60 |
| | Magnesium carbonate - - | " | 20 |
| | Pulverized sugar - - - | " | 10 |
| | Powdered soap tree bark - | " | 5 |
| | Bicarbonate of soda if indicated - | " | 5 |

To this may be added small amounts of any of the flowing essential oils. It may be made astringent by the addition of 2% of Myrrh, or antiseptic by oil of cassia or carbolic acid . No coloring matter should be added. Patient should be instructed to use this freely morning and night, using a fine bristle tooth brush which has stood in water long enough to make it pliable, or a badger's hair brush will answer. Instruct patient to take a teaspoonful of powder in the palm of the hand and after wetting the brush incorporate the powder completely into the bristles and brush every portion of the teeth, and without spitting out the powder, fill the mouth as full of water as possible, and reinsert the brush, close the lips, and wash the powder all through the teeth and appliances, holding the head over the wash basin so the escaping water may run into it. Instruct the patient to take time to do this thoroughly at least twice each day, morning and night preferably. Oxidizing powders, tooth pastes containing resinous essential oils, colored pastes and powders should not be prescribed because of their tendencies to dissolve or discolor the appliances. Our experience has been that such means can be used practically and with very good results, so far as preventing decay of the teeth or irritable conditions of the gums is concerned. The prophylaxis treatment insures clean teeth and keeps the gums and peridental tissues in the best condition for withstanding the strain of moving teeth.

Third—Can prophylaxis assist in orthodontic manipulations of teeth in adults?

It may seem that it is hardly worth while to discuss such a proposition as this, for the reason that very seldom are the orthodontists asked to interfere in cases of long standing malocclusions, or those which have been acquired in adult life, because of diseases which have affected the peridental supports of the roots, or in cases where, because of loss of some teeth, the remaining teeth have drifted into malocclusion.

The science and art of oral prophylaxis has set a higher standard for both operative and prosthetic dentistry than we have known before, and if we are not deceived in its drift, it will demand that the orthodontist of the future must extend his work to the treatment of cases of malocclusion in patients for whom such treatment has been considered impracticable. In many cases where pyorrhoea has so affected the alveolar support of the roots as to cause the teeth to assume abnormal prominence or decided malocclusion, we find that after such teeth have received prophylaxis treatment the gums and peridental conditions become healthy there is a tendency for the teeth to return to their normal positions and relations, and we believe that such teeth, if properly encouraged, would many times of themselves come back to normal relations. If they could be brought back by orthodontic force, there is no question but they would resume a good normal function and become valued organs of mastication. It may be that those of us who have been practicing oral prophylaxis have become over confident and are claiming more than we are justified in doing for this work: but when cases which seemed almost hopeless yield such beautiful results, even after a few days of treatment, and as they go on improving it gives us a greater appreciation of the value of the teeth than we have ever before had, and fills one with an enthusiastic desire to save every tooth and to bring all into a perfectly harmonious relationship such as is known only in a perfectly developed set of teeth. With the possibility of eradicated, all diseases of the structures which support the roots, and with the modern practicable crown and bridge methods of restoring lost or decaying crowns, to say nothing of the splendid attainments of the present operative procedures, it is possible to make prosthetic restorations of exceedingly high value and of the highest type of esthetic art. Is there not here an orthodontic field that would yield great reward to any one who will take up this pioneer work? Certainly no class of people need or deserve to have a complete masticatory apparatus, such as is the avowed ideal of oral prophylaxis. No one would make better use of it nor appreciate it more. Such a broadening of the scope of the orthodontists and prosthodontists would raise the professional standards of all dental practitioners and of every specialty. No one, so far as we know, has put a limit on the age where orthodontic interference should be considered impossible. If this has been done, it has been arbitrarily limited and based on experience rather than on data derived from scientific research, hence cannot be conclusive. It seems to us that age alone can never be an accurate adjustment of so important a matter. It is too much like the statement made by Dr. Osler that when men get to be sixty years old they should be chloroformed. Some men are not old at seventy, while others lose their usefulness at a much earlier age. It all depends on the man. So it is with teeth to be moved in old age or even young adult life, the decision must rest on actual conditions. We really believe that teeth can be moved at any age up to sixty years or more, providing the gums, teeth and jaws are free from disease.

If we have directed your attention to newer fields of interest and service, and you should feel disposed to enter them, we shall have done all that we could desire. We trust these few hastily prepared suggestions may commend themselves to your judgment, and that working together with the practitioner of oral porphylaxis we may thus extend our entire sphere of work and usefulness.—Reprinted from Transaction of American Society of Orthodontists, as published in *Items of Interest.*

